# Better together: An assessor support roadmap

**DOI:** 10.1111/tct.13640

**Published:** 2023-08-31

**Authors:** Wai Yee Amy Wong, Gerard J. Gormley, Sharon Haughey, Sin Wang Chong, Christine Brown Wilson

**Affiliations:** ^1^ School of Nursing and Midwifery Queen's University Belfast Belfast UK; ^2^ Centre for Medical Education, School of Medicine, Dentistry and Biomedical Sciences Queen's University Belfast Belfast UK; ^3^ School of Pharmacy Queen's University Belfast Belfast UK; ^4^ School of Social Sciences, Education and Social Work Queen's University Belfast Belfast UK; ^5^ Present address: International Education Institute University of St Andrews St Andrews UK

## 1 INTRODUCTION

Assessors, in both clinical practice and academic settings, are pivotal in making judgements on learner performance to ensure members of the public are supported by graduates who are safe and competent practitioners. However, consistency of assessor judgements of learner performance has been a concern in directly observed clinical assessments such as workplace‐based assessments (WBAs) and objective structured clinical examinations (OSCEs).^1^, ^2^ A range of sociocultural factors could influence the consistency of assessor judgements such as assessors' beliefs about the purpose of an assessment, their perception of the usefulness of the marking criteria, their expectations of learner competence and their idiosyncratic judgement practices.^3^ These inconsistencies affect the high‐stakes decisions made regarding learner progression or feedback provided that could impact their career development.

**FIGURE 1:**
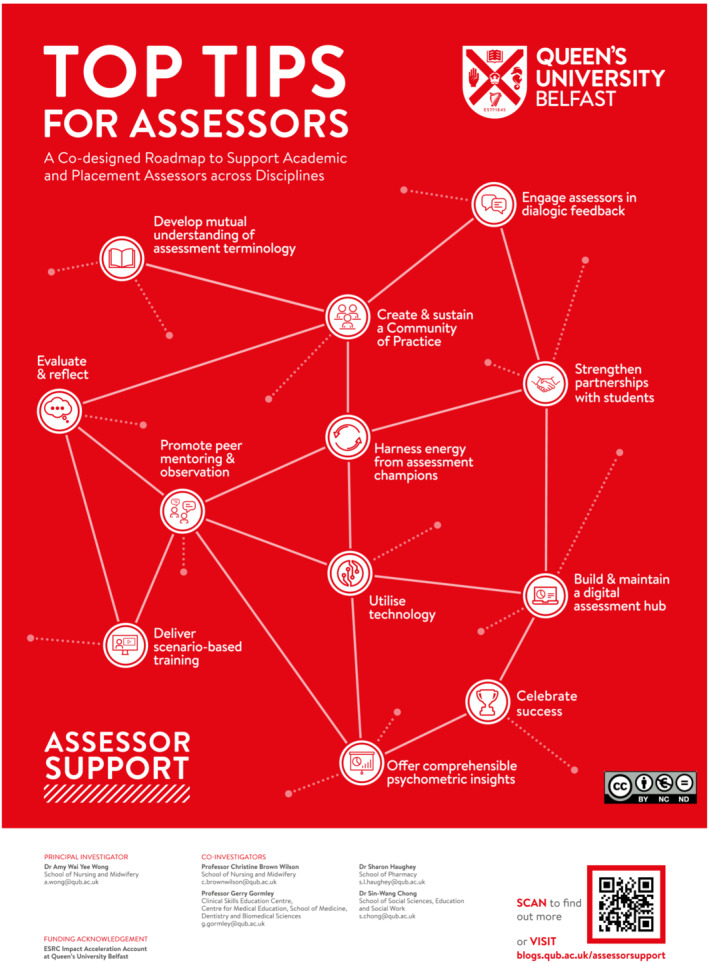
A co‐designed roadmap to support clinical and academic assessors across disciplines.^4^

Engaging assessors in faculty development could enhance their capacity and confidence in making accountable judgements. However, this presents considerable challenges, and often assessors are required to attend mandatory training with limited relevance to their professional needs. To enhance the impact of support to assessors, we need to better engage assessors with faculty development that is relevant and tailored to their professional needs. The purpose of this paper is to outline the key signposts on a faculty development roadmap in assessment^4^ (Figure  1) that are critical to consider when developing ‘fit‐for‐purpose’ faculty development initiatives. The roadmap was co‐designed with 20 assessors across medicine, nursing and midwifery, pharmacy and education and was launched in September 2021.

We need to better engage assessors with faculty development that is relevant and tailored to their professional needs.

**FIGURE 2:**
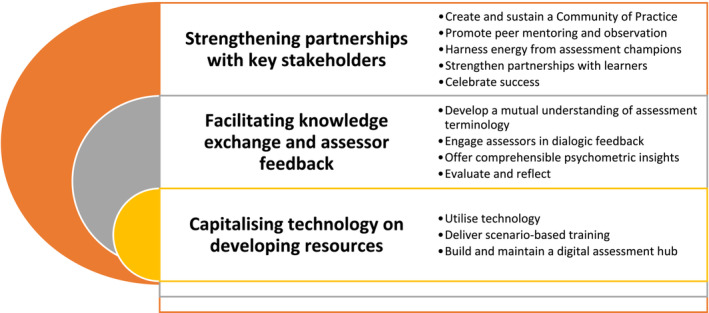
Three key areas of assessor support associated with specific signposts on the roadmap.^4^

In the following sections, we will explore three key areas of assessor support associated with specific signposts on the roadmap (Figure 2), to guide the development of ‘fit‐for‐purpose’ faculty development in assessment.

## 2 STRENGTHENING PARTNERSHIPS WITH KEY STAKEHOLDERS

### 2.1 Create and sustain a Community of Practice

A Community of Practice (CoP)^5^ acts as a link to connect, engage and build capacity among assessors who are either university academics, health practitioners or those working in both settings. Since there are common modes of assessment such as WBAs and OSCEs across health professions disciplines, an interdisciplinary CoP could further enable the learning about assessment across disciplinary boundaries. The CoP model facilitates the development of a socio‐academic network,^6^ which is fundamental to creating collective learning materials, techniques and innovation in assessments. Identifying assessment champions, together with a collective effort among assessors from different settings and disciplines, is crucial to maintaining communications to sustain an interdisciplinary CoP.

An interdisciplinary CoP could further enable the learning about assessment across disciplinary boundaries.

### 2.2 Promote peer mentoring and observation

Successful peer mentoring relies on the willingness of the mentors (experienced assessors) to share and listen to the mentees' (new assessors') concerns and provide advice relevant to their needs.^7^ The new assessors feel supported as they know there is a ‘go‐to’ person who is experienced in assessment to ask for help. When the experienced assessors provide feedback to the new assessors, they could also reflect on the challenges and rethink how these could impact their own assessment practices.

**FIGURE 3:**
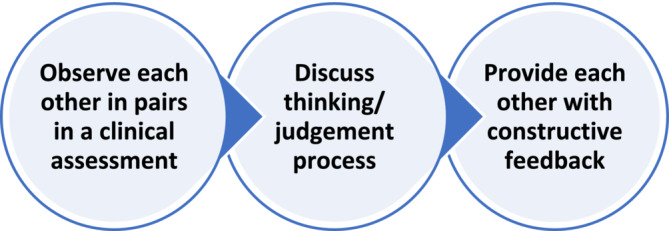
Proposed steps of peer observation of teaching in the context of a clinical assessment.

Peer observation of teaching that involves peers observing each other and providing feedback with the aim to enhance the quality of practice^8^ could also be applied in the context of assessment (Figure 3). Knowing how other assessors make their judgements in terms of the differences and similarities to one's practice could prompt reflection on areas that the assessors might have never thought of before. Peer mentoring and observation provide new and experienced assessors with the opportunity to learn from each other to better justify their judgements as part of the quality enhancement process.

### 2.3 Harness energy from assessment champions

Assessment champions play a significant role as ‘agents of change’ to drive and create changes in assessment practices. They are crucial at the start to establish the CoP but also sustain its ongoing development. These champions are the transformative agents^9^ working together to take the initiative to create novel outcomes, thus breaking away from the existing assessment practices.^10^ For example, the champions could take the lead in developing consensus among assessors on a set of accessible ‘assessment language’. They will need support from their colleagues and the institution to consolidate the suggested practices and create a collaborative change. A succession plan should also be considered to mentor the rising stars who have an interest in assessment. They bring ideas from a different perspective to maintain the enthusiasm, engagement and sustainability of transforming assessment practices to enhance the assessor and learner experience.

### 2.4 Strengthen partnerships with learners

Learners are the ones who are directly affected by the assessment outcomes and have regular interactions with the clinical assessors during their placements. Positioning learners as partners helps develop strategies that address the assessment challenges in both the university and clinical settings. Through formal recognition of the learner involvement in transforming assessment practices, it provides them with a ‘voice’ in discussing assessment without the barrier of power imbalance. Assessment practice is a developmental process more than only a judgemental process for both learners and assessors. The partnership with learners will promote the notion of a learning community in which learners are actively involved in co‐designing solutions to address the challenges in assessment.^11^

Assessment practice is a developmental process more than only a judgemental process for both learners and assessors.

Case study

Two pharmacy undergraduate students were awarded with the CW Young Fund for their summer studentships in 2022 to develop a glossary of key assessment terms for all assessors and students. They worked alongside academics, practice assessors and students from Nursing and Medicine to develop a comprehensive assessment terminology glossary. This will be used by assessors in practice settings with new placements coming on board for the new Master of Pharmacy (MPharm) degree in September 2023.

### 2.5 Celebrate success

Celebrating success is an integral part of faculty development to share ideas and strengthen collaborations. It recognises an individual or a team's achievement and builds trust and confidence among assessors, which is the foundation of enhancing the assessment experience. An annual showcase event to feature the contributions of clinical and academic assessors and highlight the success of assessor collaborations could further facilitate the connections among assessors to adapt assessment innovations in discipline‐specific and interdisciplinary assessments. Celebrating success, acknowledging assessors' contributions and sharing ideas enhance job satisfaction, expand networks and build collaborations on assessment initiatives.

## 3 FACILITATING KNOWLEDGE EXCHANGE AND ASSESSOR FEEDBACK

### 3.1 Develop a mutual understanding of assessment terminology

What are the differences between a ‘good’ and a ‘very good’ level of achievement? A mutual understanding of assessment terminology helps clarify the expected level of achievement and alleviates assessors' anxieties by knowing what they are supposed to assess the learners on. Clearly defining the competencies that are being assessed in each piece of assessment is a starting point to ensure the assessment terminology used represents the same meaning within a discipline. These competencies need to be meaningful to the assessors and learners. The mutual understanding and awareness of the shared assessment terminology will also facilitate providing feedback to learners in terms of how they can progress to the next level of achievement.

Case study

The assessment lead at the School of Nursing and Midwifery carried out a rubric development project with colleagues teaching undergraduate and postgraduate modules and a student representative using a consensus approach. The team first identified and defined a range of relevant domains: Acquisition and use of resources; Breadth/Depth of knowledge and understanding; Application of knowledge; Analysis and interpretation of data; Problem‐solving and critical thinking skills; Professionalism; Communication; and Practical skills. Then, the team created specific criteria for each level of achievement of each domain to reflect the grading boundaries. A flexible rubric template has been developed that allows module leads to select the relevant domains to create a fit‐for‐purpose rubric for an assessment task. The template enhances the clarity and transparency of the marking expectations for students and assessors, which also guides the provision of student feedback.

### 3.2 Engage assessors in dialogic feedback

Assessors are eager to receive feedback on their judgement practices to build their confidence as assessors. Providing assessors with feedback should be a two‐way communication creating an opportunity for assessors to ask questions about the written feedback and have their questions answered by experienced assessors.^12^ These dialogues should consider the contextual and individual factors to support assessors to construct meaningful interpretations of the feedback that will further enhance their assessment practices.^13^ Closing the feedback loop with assessors through dialogues is an effective way to engage assessors by recognising their contributions and encouraging self‐reflection.

These dialogues should consider the contextual and individual factors to support assessors to construct meaningful interpretations of the feedback.

### 3.3 Offer comprehensible psychometric insights

**FIGURE 4:**
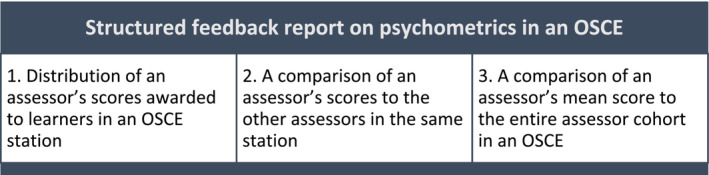
Different components of a structured feedback report on psychometrics.^14^

Psychometrics play an important role in analysing the consistency and quality assurance of scores awarded to learners. However, individual assessors' psychometric information is either often not made available to the assessors, or assessors find it challenging to interpret and apply the learning to their subsequent assessment practice. Providing OSCE assessors with a structured feedback report on psychometrics (Figure 4) could potentially reduce the assessors' variations in their stringency and leniency in the subsequent OSCE.^14^ At our institution in medicine, all OSCE examiners receive psychometric feedback on their marking for benchmarking purposes. Examiners whose pattern of marking is significantly different receive further support and training. We also collect feedback from OSCE examiners about the stations that contributes to the continuous quality improvement of the OSCEs.

To ensure that the feedback is comprehensible with the aim to encourage critical reflection on assessors' marking practice, a narrative of insights using standardised statements together with a basic individualised psychometric report is suggested to be a minimum level of feedback to assessors.

A narrative of insights using standardised statements together with a basic individualised psychometric report is suggested.

### 3.4 Evaluate and reflect

Formal and informal evaluation of assessors' marking practices could be in the form of a structured feedback report^14^ or through engaging assessors in dialogic feedback to discuss the rationale for their judgements. The feedback provides assessors with valuable information that could prompt their self‐reflection, promote critical consciousness of their abilities and initiate conversations with their mentors or peers. However, assessors need guidance on self‐reflection of their marking practices to avoid becoming overly self‐critical. They should also be aware that they might have unconsciously undertaken self‐moderation while they are marking by reflecting on the consistency of their judgements. Informal discussion between peers is also effective to identify the factors that influence assessors making objective versus intuitive judgements. It is paramount to clarify the underlying objective of the structured feedback is for faculty development and is not associated with making employment decisions.

Case study

In pharmacy, we used the shared learning from the Community of Practice set up at our institution to develop training using real, recorded examples for our assessors who were new to the OSCE assessment cycle in 2022. We then followed up with the new assessors with statistical data on their performance compared to more experienced assessors. In addition, we invited the new assessors to the OSCE development days, standard setting meetings and scrutiny panels so that they could understand fully the decision‐making process for OSCEs from start to finish. This has proven to be exceptionally useful for our new assessors, receiving positive feedback on the supportive nature of the process.

## 4 CAPITALISING TECHNOLOGY ON DEVELOPING RESOURCES

### 4.1 Utilise technology

Purposeful use of technology could enhance the efficiency of assessment processes such as digitalising feedback to learners and assessors.^15^ The assessors indicated in the co‐design workshops that their learners found the audio feedback more personal and specific to their individual assignment; therefore, they were more likely to listen to it. A short recording could be a viable alternative to provide assessors with timely feedback on their marking behaviour in an assessment task. Some of the assessors also recorded a short video to explain the expectations of a piece of assignment, and the learners found it very useful and indicated that they had repeatedly watched the recording to guide them in completing the assignment. The assessors suggested that the same will work for both clinical and academic assessors to clarify the expected learner performance in an assessment task.

Purposeful use of technology could enhance the efficiency of assessment processes such as digitalising feedback to learners and assessors.^15^

### 4.2 Deliver scenario‐based training

**FIGURE 5:**
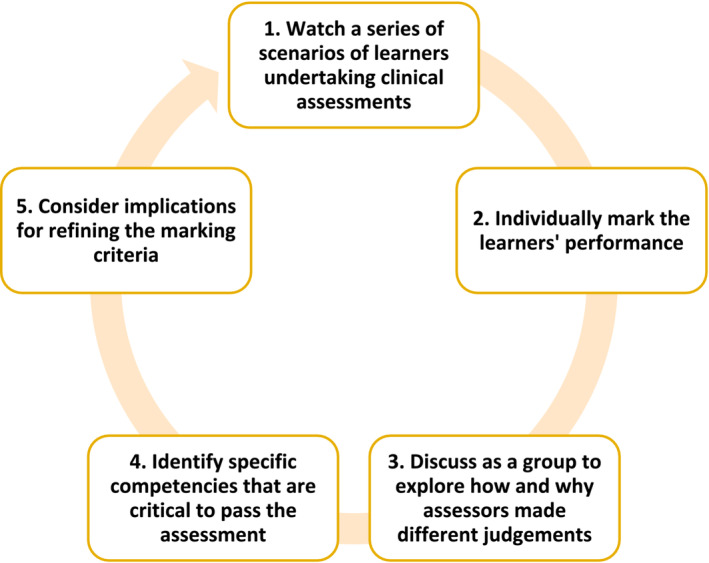
Key steps involved in scenario‐based training.

Scenario‐based training (Figure 5) could be specific to a high‐stakes assessment such as an end‐of‐programme OSCE or generic to a range of WBAs. The scenarios should also include different levels of learner performance for assessors to gain experience. The discussion is best facilitated as an interactive face‐to‐face or synchronous online session which allows assessors to ask follow‐up questions. Detailed discussion could help identify the specific competencies that are considered critical for learners to pass an assessment. The scenario‐based training will also have implications for the future development of the marking criteria or guidelines for assessors, for example, some of the key points raised in the deliberation process could be clarified by refining the marking criteria. Participation in scenario‐based training should be mandatory for new assessors. Ongoing participation is encouraged for the experienced assessors to keep well‐informed of the assessment expectations.

### 4.3 Build and maintain a digital assessment hub

Sharing knowledge, developing connections and co‐designing resources are the main purposes of building and maintaining a central digital assessment hub for assessors across disciplines. Key considerations of setting up the digital hub are its accessibility and sustainability. The digital hub must be hosted on a platform that is easily accessible by both clinical and academic assessors to minimise technical problems and to synergise the expertise of both groups. This will facilitate further collaborations among assessors such as co‐designing assessment resources. Another key point is the sustainability of the digital hub which requires a collective effort from a group of assessment champions to maintain its operation and update the information on the hub regularly. At our institution, we developed a blueprint of the digital assessment hub based on the ideas gathered in the Community of Practice. Assessors welcomed the idea of being able to access assessment resources from a central point. However, considerations need to be undertaken for the sustainability of a digital assessment hub in terms of updating information and the ongoing web hosting cost.

The sustainability of the digital hub which requires a collective effort from a group of assessment champions to maintain its operation and update the information on the hub regularly.

## 5 CONCLUSION

This paper presents a co‐designed and evidence‐guided interdisciplinary faculty development roadmap in assessment with 12 key signposts under three main areas: strengthening partnerships with key stakeholders, facilitating knowledge exchange and assessor feedback and capitalising technology on developing resources. The key signposts are the suggested starting points, which are applicable and easily adaptable across health professions disciplines to support clinical and academic assessors addressing the assessment challenges. We encourage you to consider these signposts when engaging assessors with faculty development in assessment, which could be implemented separately, or mixed and matched, to suit the contextual environment and the assessors' professional needs. There is, in fact, no end point of faculty development for assessors. The key is to sustain the selected signposts and their associated initiatives to support assessors making accountable and credible judgements of learner performance to ensure health professions graduates are safe and competent to practise.

## AUTHOR CONTRIBUTIONS

Wai Yee Amy Wong led the study conception with substantial contributions from Christine Brown Wilson, Gerard J. Gormley, Sharon Haughey and Sin Wang Chong to the design, data analysis and interpretation. Wai Yee Amy Wong wrote the first draft of the paper. All authors contributed to the critical revision of the paper and gave final approval to the submitted paper.

## CONFLICT OF INTEREST

The authors have no conflict of interest to disclose.

## ETHICAL APPROVAL

This research was approved by the Faculty of Medicine, Health and Life Sciences Research Ethics Committee (MHLS 20_129), Queen's University Belfast.

## Data Availability

Data supporting this study cannot be made available due to consent not being obtained from the participants.

## References

[1] Malau‐Aduli BS , Hays RB , D'Souza K , Smith AM , Jones K , Turner R , et al. Examiners' decision‐making processes in observation‐based clinical examinations. Med Educ. 2021;55(3):344–353. 10.1111/medu.14357 32810334

[2] Yeates P , O'Neill P , Mann K , Eva KW . Seeing the same thing differently: mechanisms that contribute to assessor differences in directly‐observed performance assessments. Adv Health Sci Educ. 2013;18(3):325–341. 10.1007/s10459-012-9372-1 22581567

[3] Wong WYA , Thistlethwaite J , Moni K , Roberts C . Using cultural historical activity theory to reflect on the sociocultural complexities in OSCE examiners' judgements. Adv Health Sci Educ. 2023;28(1):27–46. 10.1007/s10459-022-10139-1 PMC9992227 35943605

[4] Wong AWY , Brown Wilson C , Gormley G , Haughey S , Chong SW . Top tips for assessors: a co‐designed roadmap to support academic and placement assessors across disciplines. 2021.

[5] Lave J , Wenger E . Situated Learning: Legitimate Peripheral Participation Cambridge: Cambridge University Press; 1991.

[6] Saad SL , Richmond CE , Jones K , Malau‐Aduli BS . Developing a community of practice for quality assurance within healthcare assessment. Med Teach. 2021;43(2):174–181. 10.1080/0142159X.2020.1830959 33103522

[7] Donnelly R , McSweeney F . From humble beginnings: evolving mentoring within professional development for academic staff. Prof Dev Educ. 2011;37(2):259–274. 10.1080/19415257.2010.509933

[8] Sullivan PB , Buckle A , Nicky G , Atkinson SH . Peer observation of teaching as a faculty development tool. BMC Med Educ. 2012;12:26. 10.1186/1472-6920-12-26 22559217 PMC3406982

[9] Virkkunen J . Dilemmas in building shared transformative agency. Activites. 2006;3(1):43–66. 10.4000/activites.1850

[10] Engeström Y , Sannino A . Expansive learning on the move: insights from ongoing research. J Stu Educ Dev. 2016;39(3):401–435. 10.1080/02103702.2016.1189119

[11] Deeley SJ , Bovill C . Staff student partnership in assessment: enhancing assessment literacy through democratic practices. Assess Eval High Educ. 2017;42(3):463–477. 10.1080/02602938.2015.1126551

[12] Ajjawi R , Boud D . Researching feedback dialogue: an interactional analysis approach. Assess Eval High Educ. 2017;42(2):252–265. 10.1080/02602938.2015.1102863

[13] Chong SW . Reconsidering student feedback literacy from an ecological perspective. Assess Eval High Educ. 2021;46(1):92–104. 10.1080/02602938.2020.1730765

[14] Wong WYA , Roberts C , Thistlethwaite J . Impact of structured feedback on examiner judgements in objective structured clinical examinations (OSCEs) using generalisability theory. Health Prof Educ. 2020;6(2):271–281. 10.1016/j.hpe.2020.02.005

[15] Chong SW . College students' perception of e‐feedback: a grounded theory perspective. Assess Eval High Educ. 2019;44(7):1090–1105. 10.1080/02602938.2019.1572067

